# AI-Driven Design of Miniproteins as Potential Allosteric Modulators

**DOI:** 10.3390/ph19030480

**Published:** 2026-03-14

**Authors:** Xin Liu, Yunxiang Sun, Yulong Xia, Huaqiong Li, Zhiqiang Yan

**Affiliations:** 1 Zhejiang Key Laboratory of Soft Matter Biomedical Materials, Wenzhou Institute, University of Chinese Academy of Sciences, Wenzhou 325000, China; 2 School of Physical Science and Technology, Ningbo University, Ningbo 315211, China

**Keywords:** artificial intelligence, allosteric modulation, miniprotein design, de novo protein design

## Abstract

Allosteric modulation has emerged as a powerful strategy for achieving superior selectivity and safety in drug discovery and protein function regulation. Unlike highly conserved orthosteric sites, allosteric pockets are structurally diverse and less evolutionarily constrained, making them particularly suitable for modulation by designed miniproteins. Miniproteins can provide extended binding interfaces and high affinity for shallow, dynamic, or cryptic regulatory surfaces that are often inaccessible to small molecules. Recent advances in artificial intelligence (AI) are transforming this field through deep learning-based structure prediction and generative modeling. These AI-driven approaches enable the identification of allosteric hotspots, characterization of conformational ensembles, and de novo design of structured miniprotein binders. They are rapidly expanding the landscape for designing selective modulators across diverse allosteric targets, including GPCRs, receptor tyrosine kinases, nuclear receptors, ion channels, and other protein–protein interaction systems. This review summarizes state-of-the-art AI-driven computational methodologies for designing miniproteins as potential allosteric modulators and discusses their current challenges and future opportunities in allosteric drug discovery.

## 1. Introduction

**Figure 1:**
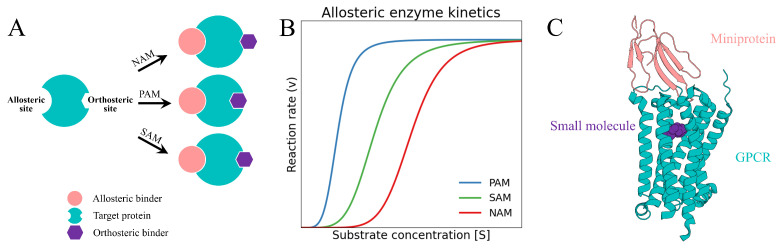
Illustrations of allosteric modulation. (**A**) Schematics of negative (NAM), positive (PAM), and silent (SAM) allosteric modulation. The allosteric binder, target protein, and orthosteric binder are shown in salmon, teal, and purple, respectively. (**B**) Allosteric enzyme kinetics showing reaction velocity versus substrate concentration for the T and R states. The R (relaxed) state exhibits higher substrate affinity and a left-shifted hyperbolic curve, whereas the T (tense) state shows lower affinity and a right-shifted curve. (**C**) A representative protein exhibiting classical allosteric modulation, illustrated by a GPCR (PDB ID: 6WJC; structure determined by X-ray crystallography at 2.55 Å resolution). The miniprotein (MT7) is highlighted in salmon, the target protein (M_1_AChR) in teal, and the small molecule (atropine) in purple.

Allosteric modulation is a fundamental mechanism of protein function in which ligand binding at a site distinct from the orthosteric pocket induces conformational or dynamic changes that modulate activity [1,2]. While orthosteric sites are usually highly conserved due to functional constraints [3], allosteric sites are more topologically diverse and less conserved, enabling greater selectivity. Several modes of allosteric modulation have been described (Figure 1A), including negative allosteric modulation (NAM), positive allosteric modulation (PAM), and silent allosteric modulation (SAM), which decrease, enhance, or lack intrinsic efficacy toward orthosteric signaling, respectively [4]. These properties make allosteric sites attractive targets for achieving improved selectivity and reduced off-target effects [4,5,6].

The molecular basis of allostery is intimately tied to protein dynamics and is best understood through the modern ensemble model [2]. It views proteins not as fixed structures but as dynamic ensembles of interconverting conformations governed by an energy landscape [7,8]. Allosteric ligands reshape this energy landscape by shifting the population distribution among conformational states. In the classical Monod–Wyman–Changeux framework [9], the conformational populations are reduced to two limiting states, the low-affinity tense (T) state and the high-affinity relaxed (R) state, whose equilibrium shift upon ligand binding gives rise to sigmoidal enzyme kinetics (Figure 1B). This population shift can occur through multiple mechanisms. These include entropic effects, kinetic coupling, and perturbations of residue interaction networks, even in the absence of observable structural changes [7]. Allosteric modulation often exploits transient or cryptic pockets that are accessible only in specific ensemble states [10]. Consequently, understanding allostery demands integrated structural, thermodynamic, and kinetic analyses. It also requires methods capable of capturing protein motions at atomic resolution across the conformational ensemble [11,12].

Allosteric modulators span diverse chemical classes. Metal ions [13] (e.g., ${\mathrm{Ca}}^{2+}$, ${\mathrm{Mg}}^{2+}$, or ${\mathrm{Na}}^{+}$) act as endogenous regulators in numerous proteins. Small molecules remain the predominant modality and have produced clinical agents targeting proteins, yet they often struggle to engage shallow, dynamic, or extended allosteric interfaces [14]. Peptides can access larger surfaces but typically suffer from limited conformational stability and poor pharmacokinetic properties [4,5]. Antibody-based binders provide high affinity and specificity at the cost of large size and restricted access to many allosteric sites [6]. Miniproteins (typically 3–8 kDa), by contrast, occupy an intermediate design space, combining larger interaction surfaces than small molecules with greater compactness and adaptability than antibodies, enabling effective recognition and modulation of complex allosteric interfaces and protein–protein interactions [6,15]. A naturally occurring example of miniprotein-mediated regulation is observed in G protein-coupled receptors (GPCRs) [16] (Figure 1C). These attributes make engineered miniproteins particularly well suited for targeting previously intractable allosteric sites, positioning them as promising scaffolds for next-generation allosteric therapeutics [17,18,19].

To date, AI-driven computational approaches are reshaping the investigation of allosteric modulation by enabling systematic analysis of complex conformational ensembles, identification of cryptic allosteric sites, and de novo design of miniproteins [20,21,22,23,24]. Miniproteins as potential allosteric modulators represent a rapidly advancing frontier, offering high specificity and enhanced interface adaptability for targets long considered “undruggable”. In the following sections, we focus specifically on AI-driven design strategies, including structure analysis and generative algorithms, and discuss how these methods are being leveraged to optimize miniprotein binders for allosteric sites with two recent and representative case studies.

## 2. AI-Driven Pipeline for Designing Allosteric Miniprotein Modulators

Miniproteins occupy a unique intermediate design space between small molecules and large protein biologics [25,26], thereby providing extended and chemically diverse interaction surfaces that are particularly well suited for engaging shallow, dynamic, and weakly conserved allosteric regulatory sites. Unlike orthosteric pockets, allosteric sites are often transient, conformationally heterogeneous, and weakly conserved, posing fundamental challenges for classical structure-based drug discovery, including the reliance on static structures that fail to capture cryptic or transiently open pockets, leading to difficulties in identifying druggable sites and achieving high-affinity binding [23,27,28].

Prior to the advent of modern AI methodologies, the computational design of miniprotein binders for allosteric modulation primarily relied on structure-guided rational design and physics-based de novo modeling [29]. These approaches leveraged experimentally determined or homology models to identify putative allosteric pockets, infer functional hotspots, and engineer binders through motif grafting, interface redesign, or scaffold repurposing. While successful in selected cases, such strategies were inherently constrained by limited conformational sampling, strong dependence on prior structural knowledge, and the difficulty of explicitly modeling long-range allosteric coupling within complex protein energy landscapes.

**Figure 2:**
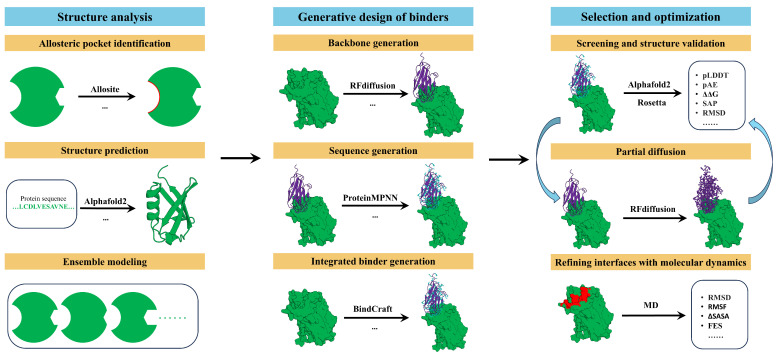
The AI-driven computational pipeline for the design of allosteric miniprotein modulators, integrating structural analysis, generative binder design and selection and optimization.

Recent advances in AI and machine learning are reshaping this design paradigm [24]. AI-driven pipelines enable a more integrated treatment of allostery by combining conformational ensemble modeling, generative backbone construction, sequence optimization, and binding assessment within a unified computational framework [30]. Collectively, AI-enabled design pipelines are transforming the development of allosteric miniprotein modulators from a largely heuristic, case-by-case endeavor into a scalable and systematic process. By coupling data-driven generative models with biophysically grounded evaluation and experimental feedback, these approaches are significantly accelerating the discovery of functional allosteric binders. In parallel, they are expanding the accessible design space beyond what was achievable with traditional computational methods alone. Figure 2 illustrates the AI-driven computational pipeline for the design of allosteric miniprotein modulators.

### 2.1. Structure Analysis

#### 2.1.1. Allosteric Pocket Identification

Identifying biologically relevant allosteric pockets is a critical first step toward understanding and engineering regulatory control in proteins [31] (Figure 2). In contrast to orthosteric binding sites, allosteric pockets are frequently shallow, weakly conserved, and highly dependent on the underlying conformational ensemble, many of which are cryptic or only transiently populated [28,32]. This intrinsic dynamical nature substantially limits the effectiveness of static, geometry-based analyses. Although experimental techniques—including NMR spectroscopy, cryo-electron microscopy, hydrogen–deuterium exchange mass spectrometry (HDX-MS), site-directed mutagenesis, and functional perturbation assays—can provide high-resolution insights into dynamic regulatory regions and allosteric communication [33,34], they are typically labor-intensive, costly, and low-throughput. These constraints have motivated the widespread adoption of computational approaches as scalable alternatives for systematic allosteric site discovery.

A broad spectrum of computational methods has therefore been developed to interrogate allostery from a dynamic and network-centric perspective [35,36,37,38,39,40,41]. Molecular dynamics simulations, Markov state models, elastic network models, coevolutionary coupling analysis, and residue interaction network theory have been routinely employed to reveal allosteric pathways, dynamic hotspots, and long-range coupling mechanisms. More recently, machine learning-based models have emerged as particularly powerful tools, leveraging curated structural and dynamical datasets to detect cryptic pockets, predict ensemble shifts, and quantify functional coupling with improved accuracy and reduced computational cost (Table 1) [42,43]. Early AI-driven approaches, such as Allosite [35], framed allosteric pocket identification as a supervised classification task using physicochemical and geometric descriptors, whereas subsequent methods, including AlloPred [36], integrated perturbation information from normal mode analysis to implicitly encode allosteric coupling. Together, these developments have established machine learning as a central paradigm for scalable and mechanistically informed allosteric pocket identification.

Further improvements were achieved by integrating richer structural feature representations and more robust learning strategies. AllositePro  [37] expanded the feature space and optimized model training to enhance prediction stability across diverse protein families. Building on curated allosteric datasets, the PASSer [38] framework adopted ensemble learning to improve generalization and scalability, while PASSer 2.0 [39] further advanced this direction through an AutoML architecture that automates feature selection and model optimization. Rather than treating allosteric pocket identification as a binary classification task, PASSerRank [40] reframed the problem as a learning-to-rank task, enabling prioritization of candidate pockets according to their predicted regulatory relevance. This approach achieved superior performance, ranking known allosteric pockets in the top 3 positions for 83.6% (ASD dataset) and 80.5% (CASBench) of test proteins, with higher F1 scores (0.662 on ASD, 0.608 on CASBench) and Matthews correlation coefficients (0.645 on ASD, 0.589 on CASBench) compared to conventional classifiers.

Most recently, ensemble optimization strategies such as MEF-AlloSite [41] have combined multiple machine learning models with optimized feature selection from thousands of pocket descriptors to achieve improved robustness and accuracy in identifying allosteric regions at both pocket and site levels. Compared to state-of-the-art methods like PASSer 2.0 and PASSerRank, MEF-AlloSite demonstrated statistically significant gains (1–6% higher mean average precision across multiple test sets, with *p* < 0.05 and Cohen’s d > 0.5), along with enhanced classification accuracy (ranging from 0.452 to 0.620 on varied test cases). These AI-driven approaches transform allosteric pocket identification from heuristic, structure-centric analyses into a data-driven inference problem, providing a systematic and scalable foundation for downstream allosteric modulator and binder design.

#### 2.1.2. Structure Prediction and Ensemble Modeling

Accurate structural models of target proteins are critical for designing allosteric miniprotein binders, as allosteric modulation and ligand recognition are governed by protein dynamics and conformational ensembles rather than a single static structure [44]. Allosteric modulation is fundamentally an ensemble phenomenon, operating through shifts in conformational equilibria on a complex energy landscape, which makes ensemble-level structural information essential for rational binder design [1,2,8]. Traditional experimental structures obtained by X-ray crystallography or cryo-EM typically represent low-energy or highly populated conformations and may fail to capture excited or low-population states that harbor functional allosteric or cryptic binding sites [14,32].

Recent advances in AI-based and MSA-based structure prediction have substantially expanded access to high-quality atomic models (Table 2). Deep learning frameworks such as AlphaFold and RoseTTAFold achieve near-experimental accuracy for monomeric proteins and many protein complexes by leveraging evolutionary information encoded in multiple sequence alignments [45,46,47]. Extensions including AlphaFold-Multimer and AlphaFold 3 further enable modeling of oligomeric assemblies and biomolecular interactions that are directly relevant to allosteric signaling and regulation, with some predictions showing agreement with experimental structures of allosteric proteins and ligand-binding sites [48]. These AI-predicted structures often serve as starting points for exploring conformational variability and for generating structural hypotheses in systems lacking experimental data, although they remain speculative and require experimental validation, particularly in dynamic allosteric systems [24,29].

To address the inherently dynamic nature of allostery, structure prediction is increasingly integrated with ensemble modeling techniques [12,49,50]. Molecular dynamics simulations, together with enhanced sampling approaches such as metadynamics or replica-exchange molecular dynamics, enable systematic exploration of conformational landscapes beyond single predicted structures and provide access to transient or functionally relevant states [2,43]. AI-assisted strategies further guide ensemble generation by biasing sampling toward alternative conformational states inferred from evolutionary couplings, energetic frustration, or learned structure–dynamics relationships [10,22].

Such ensemble models are particularly valuable for miniprotein binder design, as they help identify conformations that expose regulatory surfaces compatible with extended protein–protein interaction interfaces [25,26]. In practical design workflows, ensemble-aware structural models guide the selection of target conformations for binder generation, reducing the risk of designing binders that recognize only rare or non-functional states and improving the likelihood of functional allosteric modulation. Recent AI-driven binder design frameworks explicitly benefit from this ensemble perspective, linking structure prediction and conformational sampling with generative design strategies [17,30].

**Table 2:** Representative MSA-based AI tools for protein structure prediction and validation.

Tool ^a^	Method and Key Features	Year	Ref.
trRosetta	Deep learning model predicting inter-residue distances and orientations from MSA-derived features; early high-throughput deep predictor for fold inference.	2020	[47]
RoseTTAFold	Three-track neural network integrating sequence, pairwise distances, and 3D coordinates; uses MSAs for accurate monomer and multimer predictions.	2021	[46]
AlphaFold2	Deep learning model using MSA and Evoformer architecture; delivers high-accuracy monomer and complex structure predictions with confidence metrics.	2021	[45]
AlphaFold-Multimer	Extension of AlphaFold2 for protein complex modeling; incorporates paired MSAs to capture inter-chain co-evolutionary signals.	2022	[51]
AlphaFold3	Diffusion-based generative architecture for modeling protein–ligand, protein–DNA, and protein–protein complexes, while still using MSA information.	2024	[52]

^a^ These tools rely primarily on multiple sequence alignment (MSA) to capture evolutionary and co-evolutionary constraints, and have been widely used for structural validation in binder design workflows.

### 2.2. Generative Design of Binders

Following structural analysis, the generative design stage focuses on constructing miniprotein binders that are compatible with target allosteric sites in terms of geometry, energetics, and dynamics. Unlike small-molecule design, which primarily emphasizes pocket occupancy, miniprotein design seeks to engineer extended protein–protein interfaces. These interfaces are intended to engage regulatory surfaces and stabilize specific conformational states along the target protein’s energy landscape. Accordingly, generative models are required not only to produce binders with high affinity but also to shape interactions that bias conformational equilibria underlying allosteric modulation [53].

AI-driven generative frameworks treat binder design as a conditional generation problem [54]. In this setting, backbone topology and amino acid sequences are sampled under explicit constraints defined by the target structure, binding geometry, and functional objectives (Figure 2). These pipelines typically decompose the design task into backbone generation, sequence design, and integrated co-optimization steps. Such modularization enables efficient exploration of the vast combinatorial space associated with miniprotein binders (Table 3).

#### 2.2.1. Backbone Generation

Backbone generation represents the first and most structurally constrained stage of de novo miniprotein binder design, in which three-dimensional protein scaffolds are generated to geometrically complement a target binding surface. At the miniprotein scale, backbone topology critically determines fold stability, surface curvature, and the spatial organization of interface residues. Consequently, backbone generation models must balance physical realism with sufficient flexibility to accommodate diverse protein–protein interaction geometries.

Recent advances in this area have been driven primarily by generative models operating directly in three-dimensional coordinate space, with diffusion- and flow-based approaches emerging as dominant paradigms [55,56,57,58]. RFdiffusion exemplifies diffusion-based backbone generation, formulating scaffold design as an iterative denoising process that transforms random coordinate noise into structured protein backbones consistent with learned geometric priors [55]. By conditioning generation on target surface geometry, interface residue positions, or rigid-body constraints, RFdiffusion enables the design of miniprotein backbones that are explicitly shaped to engage challenging protein surfaces, including shallow and discontinuous allosteric regions.

In contrast, complementary generative models such as Chroma [59] and FoldFlow [58] primarily focus on general de novo protein backbone generation rather than target-conditioned binder design, and thus currently offer limited direct utility for high-precision binder scaffolding, despite their potential as future extensible frameworks. These backbone-generation methods aim to produce physically plausible and foldable miniprotein scaffolds that define a viable structural substrate for downstream optimization. However, backbone-only generation does not ensure functional binding, as interface chemistry and energetic complementarity are not explicitly resolved at this stage. Backbone generation therefore serves primarily to establish geometric feasibility, while sequence design and integrated structure–sequence optimization are required to achieve functional miniprotein binders.

#### 2.2.2. Sequence Design

Following backbone generation, sequence design assigns amino acid identities that stabilize the intended fold and mediate favorable interactions with the target surface. This inverse folding problem is particularly stringent for miniprotein binders, where limited sequence length balances the coupling between folding stability, interface specificity, and conformational robustness. AI-driven sequence design approaches address this challenge by learning conditional probability distributions over sequence space given a fixed three-dimensional backbone.

Graph-based neural architectures trained on large structural datasets have become central to AI-driven sequence design. ProteinMPNN [60] represents a widely adopted message-passing neural network that encodes residue–residue spatial relationships and predicts amino acid probabilities compatible with a given backbone, enabling efficient and accurate sequence optimization for miniprotein scaffolds. In addition to computational benchmarks, ProteinMPNN-designed sequences have been experimentally validated in functional allosteric modulation contexts, such as redesign of ubiquitin variants that bind and allosterically activate Rsp5 E3 ligase in vitro [61]. These studies support the utility of ProteinMPNN for generating sequences that not only fold correctly but also exert measurable regulatory effects on target proteins. PiFold [62] adopts a related graph neural network paradigm optimized for scalability and computational efficiency, facilitating rapid sequence design cycles for compact proteins.

An alternative class of inverse folding models leverages pretrained protein language models. ESM-IF1 [63] projects structural information into semantically enriched embedding spaces learned from large-scale sequence data, implicitly encoding evolutionary and biophysical constraints. This language-model-based strategy enables the generation of sequences that are not only structurally compatible but also evolutionarily plausible.

Across these approaches, the unifying objective is to generate sequences that reliably fold into the designed backbone while forming specific, energetically favorable contacts at the binder–target interface. Because these models typically assume a fixed backbone, their effectiveness motivates the development of integrated frameworks that jointly optimize structure and sequence, as introduced in the following.

#### 2.2.3. Integrated Binder Generation

While backbone generation and sequence design can be executed as separate stages, integrated binder generation frameworks aim to co-optimize structure and sequence within a unified generative process. This integration is particularly advantageous for miniprotein binders, where the interface geometry, residue composition, and folding stability are tightly coupled.

Several integrated AI-based approaches relying on structure prediction feedback have guided generative optimization. AlphaProteo (2024) [64] and AlphaDesign (2025) [65] employ AlphaFold-assisted evaluation to iteratively refine binder sequences and conformations toward structurally stable and functionally competent states. In these frameworks, structure prediction acts as an implicit physical filter that biases generative sampling toward favorable folding and interaction profiles. O-design [66] emphasizes objective-driven interface refinement by combining energy-based scoring with deep learning–guided sequence optimization.

Some other recent methods adopt fully end-to-end generative paradigms. BindCraft [67] implements an automated one-shot design pipeline that integrates backbone generation, sequence assignment, and confidence-based filtering, achieving experimental hit rates of 10–100% for de novo miniprotein binders across diverse targets (e.g., 13/53 for PD-1, 7/9 for PD-L1, and 4/16 for CD45). BoltzGen [68] extends this concept through an all-atom generative framework that unifies structure and sequence sampling, enabling universal binder design across diverse protein targets, with wet-lab validation yielding nanomolar-affinity binders for 66% of nine novel targets (for both nanobody and general protein designs). Similarly, PXDesign [69] provides an end-to-end pipeline combining generative modeling with rigorous post hoc confidence assessment to prioritize experimentally viable designs.

PPDiff [70] represents a joint sequence–structure diffusion framework capable of directly generating protein–protein complexes, including miniprotein binders, within a single generative process. By modeling interface formation as an emergent property of coupled structure and sequence generation, such approaches further blur the boundary between backbone synthesis and sequence design.

Together, these integrated generative systems represent a significant advance in computational binder design, enabling coherent exploration of structure–sequence space and providing scalable, AI-driven routes to engineer high-affinity and high-specificity miniprotein binders for challenging protein targets.

**Table 3:** Key AI Methods for Miniprotein Binder Design.

Category	Tool	Core Capability	Year	Ref.
Backbone generation	**RFdiffusion**	Diffusion-based backbone generation conditioned on target interfaces for stable miniprotein scaffolds	2023	[55]
Sequence generation	**ProteinMPNN**	Inverse folding-based sequence design for fixed backbone miniproteins	2022	[60]
	**ESM-IF1**	Protein language model-based inverse folding for sequence design on fixed miniprotein backbones	2022	[63]
	**PiFold**	Graph neural network-based inverse folding enabling efficient miniprotein sequence design	2022	[62]
Integrated design of backbone and sequence	**AlphaProteo**	AlphaFold-assisted binder design emphasizing functional interaction motifs	2024	[64]
	**BindCraft**	Automated one-shot de novo miniprotein binder design with high experimental hit rates	2024	[67]
	**O-design**	Objective-driven interface refinement via energy-based and deep learning-assisted sequence optimization	2025	[66]
	**AlphaDesign**	AlphaFold-guided hallucination with diffusion-based sequence optimization for multistate binder design	2025	[65]
	**BoltzGen**	All-atom generative model unifying structure and sequence for universal binder design, including miniproteins	2025	[68]
	**PXDesign**	End-to-end de novo binder design pipeline (generation plus confidence filtering) with high experimental success rates	2025	[69]
	**PPDiff**	Joint sequence–structure diffusion framework for direct generation of protein–protein complexes and miniprotein binders	2025	[70]

### 2.3. Selection and Optimization

#### 2.3.1. Screening and Structure Validation

After generative modeling, large numbers of de novo miniprotein binders must be computationally screened to identify candidates that are structurally reliable and likely to engage the target (Figure 2) [71]. In this stage, MSA-based structure prediction models, particularly AlphaFold2, serve as high-precision filters. Although these binders typically lack natural evolutionary homologs, AlphaFold’s implicit structural and physical priors provide stringent checks on foldability and topology. Key screening metrics include the predicted Local Distance Difference Test (pLDDT), which estimates residue-level structural confidence, and predicted aligned error (pAE), which evaluates the relative positioning of residue pairs across the binder–target interface [72]. Designs with low pLDDT, high interface pAE, or significant deviations from the intended backbone (e.g., measured via RMSD) are efficiently filtered, ensuring that only geometrically plausible candidates proceed to downstream validation.

Complementary to AI-driven screening, physics-based scoring functions, most commonly implemented in Rosetta, assess atomic-level interactions and interface quality. The Rosetta interface binding free energy change (ΔΔG) estimates the energetic contribution of the binder–target interface, while surface complementarity and hydrophobic packing are evaluated using metrics such as the solvent-accessible surface area penalty (SAP_score) [73]. Integrating AlphaFold2 confidence metrics with Rosetta energy-based evaluations provides a balanced assessment of both structural plausibility and interface competency [18,19]. Designs that satisfy both criteria are prioritized as high-confidence candidates for experimental characterization, ensuring that selected binders are geometrically consistent, energetically favorable, and likely to function as intended.

#### 2.3.2. Partial Diffusion

Partial diffusion is a refinement technique in computational protein design, implemented within the RFdiffusion framework [55]. It works by selectively adding noise to certain regions of a protein structure and then denoising them to generate optimized variations, while keeping other regions fixed. In the context of miniprotein binder design, this approach enhances affinity, diversity, and specificity. RFdiffusion can selectively re-diffuse only a subset of high-ranked backbones, preserving key structural elements such as the overall fold or anchoring interactions at the allosteric site. This targeted resampling enables local structural optimization without disrupting previously identified favorable binding geometries.

Following partial diffusion, redesigned backbones are subjected to sequence redesign, and the resulting models are re-evaluated using AlphaFold2 confidence metrics, including pLDDT and interface pAE. Designs that exhibit improved structural confidence or interface definition are retained and recycled as inputs for subsequent rounds of partial diffusion. By iterating this cycle of constrained backbone resampling, sequence optimization, and AlphaFold2-based evaluation, the overall quality and reliability of allosteric miniprotein designs can be progressively enhanced [30,74].

#### 2.3.3. Refining Interfaces with Molecular Dynamics

Molecular dynamics (MD) simulations complement static structure predictions by explicitly sampling the conformational ensemble of protein–protein complexes, allowing assessment of interface stability and flexibility under near-physiological conditions. Unlike single snapshot models, trajectory-based MD reveals transient fluctuations at the binding interface and helps identify regions that maintain key contacts versus those that undergo rearrangements, thus providing a physics-based check on conformational plausibility. Such dynamic insights are particularly valuable for the design of miniprotein binders or small-molecule protein–protein interaction (PPI) inhibitors, where maintaining precise interfacial contacts is critical for functional inhibition [75].

Beyond qualitative assessment, MD trajectories can be integrated with post-processing methods, such as clustering representative conformations, estimating relative free energies, or re-scoring with ensemble-based metrics, to refine predicted complexes toward more accurate interfacial geometries and energetics. By combining initial AI-driven design models with MD-derived ensembles, researchers can prioritize candidates with both robust dynamic stability and favorable interaction profiles, facilitating the development of effective miniprotein binders or PPI inhibitors for experimental validation [76].

## 3. Latest Case Study in AI-Driven Design of Miniprotein Modulators

**Figure 3:**
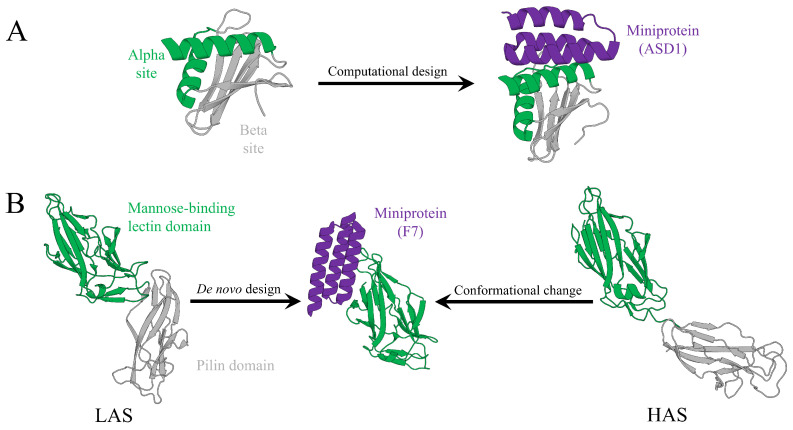
Representative case studies in AI-driven design of miniprotein modulators, illustrating the targeting of non-orthosteric surfaces and the mechanisms underlying conformational modulation with two examples. (**A**) A miniprotein binder (ASD1) designed against the α site of the Flpp3 virulence factor, with the α site and β site shown in green and gray, and the designed miniprotein shown in purple, respectively. (**B**) A miniprotein inhibitor (F7) targeting the mannose-binding lectin domain of FimH; F7 can bind both the high-affinity (HAS) and low-affinity (LAS) conformations and, upon binding to the HAS, induces a conformational transition toward the LAS, with the lectin domain and pilin domain shown in green and gray, and the designed miniprotein shown in purple, respectively.

While direct precedents for AI-driven miniprotein design explicitly targeting allosteric sites remain limited, two recent studies employing de novo design against non-orthosteric pockets provide close analogs. These two approaches modulate protein function through conformational perturbations or interface disruptions, akin to allosteric mechanisms. Below, we introduce these two representative examples (Figure 3 and Table 4).

### 3.1. Case 1: High-Affinity Binders to the Flpp3 Virulence Factor

A compelling example is the de novo design of high-affinity miniprotein binders targeting Flpp3, a virulence factor from *Francisella tularensis* (Figure 3A) [77]. Notably, Flpp3 lacks deep pockets or known binding partners, rendering it challenging for conventional small-molecule inhibition. The designed binders aim to target two distinct surfaces on Flpp3. Site I corresponds to an electronegative α-helical face that is hypothesized to mediate membrane interaction, whereas Site II is an electropositive β-sheet face. Binding at these surfaces may disrupt immune evasion and bacterial dissemination through induced conformational changes or modulation of protein–protein interactions. Although not explicitly described as allosteric, this strategy closely parallels classical allosteric modulation.

The design pipeline integrated physics-based docking with deep learning tools. Rotamer interaction fields were generated using RIFGen, scaffold placement was performed with PatchDock, and interface refinement was carried out using RIFDock. ProteinMPNN was employed for sequence design, followed by iterative backbone optimization using Rosetta FastRelax. AlphaFold2 was then used for model filtering. Selection criteria emphasized predicted structural confidence and interface quality, including pLDDT values greater than 80–90, interface pAE below 6, Rosetta ΔΔG values less than −35 to −40 kcal/mol, and SAP scores below 30–35.

The pipeline began with a library of 43,724 miniprotein scaffolds ranging from 25 to 65 amino acids in length and generated approximately 500,000 docked conformations. This process yielded 15,000 α-site and 8817 β-site candidates for experimental screening. Experimental screening and validation confirmed that several designed miniproteins bind *Flpp3* with nanomolar to sub-nanomolar affinity. Yeast surface display–based selection identified a small set of enriched α- and β-site binders adopting three-helix bundle topologies, consistent with the intended scaffold designs.

Biophysical characterization demonstrated high binding affinity and exceptional stability of the top candidates, while structural analyses revealed near-atomic agreement between the designed models and experimentally determined complex structures. These results validate the accuracy of the AI-assisted design pipeline in targeting shallow, non-orthosteric protein surfaces and demonstrate its potential for functional modulation through allosteric-like mechanisms.

### 3.2. Case 2: Miniprotein Inhibitors of Bacterial Adhesins

Another illustrative example is the design of miniprotein inhibitors targeting chaperone–usher pathway (CUP) adhesins from uropathogenic *Escherichia coli* and *Acinetobacter baumannii*, which mediate urinary tract infections (UTIs) (Figure 3B) [78]. Here, we focus on designed F7, a miniprotein inhibitor of the FimH adhesin from *E. coli*. Rather than directly occupying the orthosteric host receptor-binding site, F7 binds to a pocket adjacent to this site that is preferentially accessible in the low-affinity state (LAS) of FimH. Binding at this site induces a conformational population shift that disfavors the high-affinity state (HAS), thereby exerting allosteric-like inhibitory effects. By stabilizing inactive conformations, F7 disrupts host receptor binding and biofilm formation.

F7 was wholly designed using the AI-driven pipeline to explore the large conformational and sequence space, integrating hotspot-conditioned RFdiffusion, ProteinMPNN sequence optimization, Rosetta interface evaluation, and AlphaFold2 (AF2)-based structural filtering. Approximately 10,000 backbone designs were generated per target using crystal structures of FimH in both the high-affinity (HAS; PDB: 1UWF) and low-affinity (LAS; PDB: 3JWN) states, with hotspot residues proximal to the mannose-binding pocket specified as diffusion constraints. For each backbone, multiple sequences were assigned using ProteinMPNN and subjected to initial AF2 filtering, retaining designs with pLDDT values greater than 80 and predicted aligned error (pAE) below 10.

High-confidence designs were further refined through iterative partial diffusion, followed by sequence reassignment and AF2 evaluation. Final candidates were selected using stringent complex- and monomer-level criteria, including AF2 metrics for the complex (binder pLDDT ≥ 90 and interface pAE ≤ 6–6.5), favorable Rosetta interface metrics (ΔΔG≤−30 kcal/mol and SAP score ≤ 40), and AF2 monomer confidence for the isolated minibinder (pLDDT ≥ 90). Top-ranked designs were synthesized and screened by cDNA display, leading to the identification of F7.

Experimental screening and validation identified F7 as a high-affinity binder that selectively stabilizes the low-affinity state of FimH, inhibiting red blood cell aggregation and biofilm formation. Structural and functional analyses, including X-ray crystallography, NMR spectroscopy, and in vivo UTI models, confirmed the designed binding mode and conformational modulation mechanism.

## 4. Discussion

Despite the rapid progress of artificial intelligence in protein structure prediction and generative design, several important limitations remain in designing miniproteins as potential allosteric modulators.

First, current AI-based structure prediction frameworks, exemplified by AlphaFold-class models, primarily generate a single high-confidence static structure rather than the full conformational ensemble that proteins populate under physiological conditions [20,52]. This representation is fundamentally mismatched with the intrinsically dynamic nature of proteins and therefore limits direct access to folding pathways, energy landscapes, and low-population but functionally relevant conformational states. Training datasets are also heavily biased toward crystallographic and cryo-EM structures, which often capture stabilized or experimentally trapped conformations. As a result, flexible segments and intrinsically disordered regions are frequently assigned low confidence or poorly defined structures despite their central roles in allosteric communication. In addition, current predictors cannot explicitly distinguish ligand-free and ligand-bound conformational preferences, making it difficult to capture induced-fit or conformational-selection mechanisms or to describe how allosteric perturbations reshape the underlying energy landscape [79].

Second, accurate prediction of allosteric pockets and communication pathways remains challenging. Although AI integrated with molecular simulations has improved the identification of potential regulatory sites, predictions still suffer from substantial false positives and misannotations, and experimental validation remains necessary [21]. Moreover, most approaches focus on static pocket detection and lack mechanistic insight into long-range energy propagation or population shifts within conformational ensembles. Predicting allosteric communication networks often requires extensive molecular dynamics simulations, which remain computationally demanding and limit large-scale application [20].

Third, limitations also exist in AI-driven generative frameworks for the design of de novo miniprotein binders. Current scoring models typically learn from datasets of binding energies or structural metrics and may overfit to known complexes, resulting in limited generalization to unseen folds, scaffolds, or functional interfaces [53,60]. Consequently, predicted scores do not always correlate well with experimental binding affinity or kinetics, and different computational models may produce inconsistent rankings.

Furthermore, although generative models can efficiently propose candidate sequences and backbones, many computationally designed structures fail to fold correctly, express efficiently, or form stable complexes with their targets during experimental validation [53]. The design of novel topologies or previously unobserved interface geometries further challenges model generalization. Evaluation metrics used in current pipelines—including ML-based scores, energy functions, and structure prediction confidence—remain imperfect proxies for experimental success. As a result, the wet-lab success rate of de novo binders is often low (typically <20%), requiring extensive experimental screening [67,68].

Overall, these challenges highlight the need for improved ensemble-aware modeling of protein dynamics, more accurate prediction of allosteric communication mechanisms, and enhanced scoring and validation frameworks that integrate computational design with iterative experimental feedback [71].

## 5. Conclusions and Future Prospects

Allosteric modulation represents a fundamental principle by which biological systems achieve long-range control over protein activity, signal transduction, and gene regulation. Proteins do not function solely through isolated active sites; rather, they operate as dynamic networks, where local perturbations propagate across spatial distances to modulate enzymatic activity, receptor signaling, oligomerization, and transcriptional output. This capacity for remote control underlies critical biological processes, including ion channel gating, receptor activation, chromatin remodeling, and transcriptional regulation, and is thus considered a “second layer” of regulation beyond primary ligand recognition, providing robustness and tunability to complex signaling systems [80].

From a therapeutic perspective, allosteric modulation offers significant advantages over conventional orthosteric drugs. Orthosteric sites are often highly conserved, particularly within kinases, GPCRs, and nuclear receptors, increasing the risk of off-target binding, cross-reactivity, and dose-limiting toxicity. Even highly specific orthosteric ligands may induce adverse effects by perturbing normal physiological signaling. In contrast, allosteric modulators act at less conserved regulatory sites, enabling higher selectivity, reduced systemic toxicity, and precise modulation of protein function rather than simple inhibition or activation [27,81,82,83]. By modulating conformational equilibria, allosteric binders can bias signaling pathways, control oligomeric states, or selectively affect disease-associated functional states, thus expanding the druggable target space.

Despite these advantages, allosteric modulators have historically been challenging to discover [4,6]. Allosteric sites are often shallow, transient, or only populated in specific conformational states, making them poorly suited to traditional high-throughput screening strategies optimized for small molecules. A lack of structural and mechanistic understanding of long-range coupling further limits rational intervention. Recent advances in cryo-electron microscopy, solution-based structural techniques, and the accumulation of large structural databases have begun to illuminate these regulatory landscapes. Nonetheless, experimental approaches alone are insufficient to systematically explore the vast combinatorial space of potential allosteric binders.

In this context, AI-driven protein design has emerged as a transformative enabling technology. By learning from large-scale structural, sequence, and biophysical data, modern AI models capture the principles governing protein folding, interaction geometry, and conformational plasticity. Unlike traditional optimization strategies that primarily refine existing scaffolds, AI enables genuine de novo exploration of protein sequence and structure space, allowing the design of entirely new architectures tailored to specific regulatory sites. Importantly, AI does not replace experimental screening but refines it: computational design narrows the astronomically large sequence space to a high-quality subset, efficiently guiding subsequent experimental selection and optimization.

Within this paradigm, miniproteins occupy a uniquely advantageous position [15,17,18,19,77,78]. Compared to small molecules, they offer larger and more versatile interaction surfaces, enabling high-affinity and highly selective recognition of shallow or extended allosteric interfaces. Their inherent structural diversity and capacity for multivalent interactions allow effective engagement of regulatory surfaces involved in protein–protein interactions and conformational control. At the same time, miniproteins are smaller than antibodies, allowing access to sterically restricted environments and, in some cases, intracellular targets inaccessible to large biologics. Advances in computational stabilization and sequence optimization further enhance their structural robustness and functional reliability, making them ideal candidates for allosteric modulation. Models such as RFdiffusion [55] enable de novo generation of backbone architectures compatible with target regulatory surfaces, while ProteinMPNN [60] provides efficient sequence optimization for stability and interface complementarity. These tools allow rational construction of novel miniprotein scaffolds tailored to specific allosteric pockets, rather than relying on incremental modification of existing proteins.

Taken together, AI-driven de novo design of miniprotein allosteric modulators represents not merely an incremental improvement but a qualitative shift in drug discovery strategy. Rather than competing with endogenous ligands at highly conserved active sites, this approach leverages regulatory architecture, conformational dynamics, and system-level control to achieve precise modulation of protein function. While challenges remain—including accurate modeling of conformational entropy, reliable prediction of in vivo behavior, and integration of developability constraints—the convergence of AI, structural biology, and protein engineering provides a powerful framework to address them.

Ultimately, as AI-driven design methodologies continue to mature, this paradigm promises to deepen mechanistic understanding of allosteric modulation while enabling novel therapeutic strategies. By coupling de novo protein design with iterative experimental validation, it offers not only new avenues for drug development but also a framework for probing how biological systems encode and transmit regulatory information across molecular scales [84].

## Figures and Tables

**Table 1:** Machine learning-based tools for allosteric pocket identification. The tools were evaluated on different datasets as reported in their original studies.

Tool ^a^	Machine-Learning Strategy and Key Features	Year	Ref.
Allosite	Support vector machine classifier trained on static structural descriptors to discriminate allosteric from non-allosteric pockets	2013	[35]
AlloPred	Perturbation-guided machine learning scoring of candidate pockets combined with normal mode analysis	2016	[36]
AllositePro	Structure-based machine learning framework integrating multiple physicochemical and geometric features for improved robustness	2017	[37]
PASSer	Ensemble machine learning approach trained on curated allosteric datasets for large-scale pocket identification	2021	[38]
PASSer 2.0	AutoML-driven framework enabling automated feature selection, model optimization, and improved generalization	2022	[39]
PASSerRank	Learning-to-rank strategy for prioritizing predicted allosteric pockets rather than binary classification	2023	[40]
MEF-AlloSite	Multi-model ensemble learning with optimized feature selection for accurate identification of allosteric sites and pockets	2024	[41]

^a^ We include only machine learning-based methods explicitly developed for allosteric pocket identification or prioritization, excluding approaches aimed at inferring allosteric signaling pathways, identifying key allosteric residues, or relying on non-machine learning-based prediction strategies.

**Table 4:** Experimental results from AI-driven miniprotein design cases. This table summarizes key outcomes from two representative case studies, highlighting the functional and therapeutic impact of the designed miniproteins.

Category	Case 1: Flpp3 Binders	Case 2: FimH Inhibitor (F7)
**Target protein**	Flpp3 virulence factor from *Francisella tularensis*	FimH adhesin from uropathogenic *Escherichia coli*
**Binding site **	Site I (α-helical face, membrane interaction) and Site II (β-sheet face); allosteric-like disruption of protein–protein interactions	Pocket adjacent to the orthosteric site; stabilizes the low-affinity state (LAS) and disfavors the high-affinity state (HAS), leading to allosteric inhibition
**Binding affinity **	Site I: 24–110 nM; Site II: initially 81 nM, optimized to sub-nanomolar range	Nanomolar affinity confirmed by screening; selectively stabilizes LAS
**Structural validation **	Circular dichroism confirms three-helix bundle topology; X-ray crystal structure RMSD of 0.9 Å relative to the design model	X-ray crystallography confirms the binding mode; NMR spectroscopy validates the induced conformational shift
**Functional effects **	High stability; disrupts immune evasion, bacterial dissemination, and plasminogen binding; validated by yeast surface display enrichment and biolayer interferometry	Inhibits red blood cell aggregation, biofilm formation, and host receptor binding; blocks pathogenesis without direct orthosteric competition
**Practical impact/in vivo results **	Provides research tools to probe Flpp3 function in tularemia and supports development of therapeutic candidates against antibiotic-resistant strains	Effective in treating and preventing uncomplicated and catheter-associated UTIs in mouse models, representing an antibiotic-sparing strategy for MDR infections

## Data Availability

No new data were created or analyzed in this study. Data sharing is not applicable.
